# The Role of Vitamin D Receptor Gene Polymorphisms in Colorectal Cancer Risk

**DOI:** 10.3390/cancers12061379

**Published:** 2020-05-27

**Authors:** Ippokratis Messaritakis, Asimina Koulouridi, Maria Sfakianaki, Konstantinos Vogiatzoglou, Nikolaos Gouvas, Elias Athanasakis, John Tsiaoussis, Evangelos Xynos, Dimitriοs Mavroudis, Maria Tzardi, John Souglakos

**Affiliations:** 1 Laboratory of Translational Oncology, Medical School, University of Crete, 70013 Heraklion, Greece; asi_minakoulouridi@yahoo.com (A.K.); mimasf19@gmail.com (M.S.); vogiatzogloukwstas@gmail.com (K.V.); mavroudis@uoc.gr (D.M.); johnsougl@gmail.com (J.S.); 2 Medical School, University of Cyprus, 20537 Nicosia, Cyprus; nikos.gouvas@gmail.com; 3 Department of General Surgery, Heraklion University Hospital, 71110 Heraklion, Greece; eliasathanasakis@yahoo.gr; 4 Department of Anatomy, School of Medicine, University of Crete, 70013 Heraklion, Greece; tsiaoussis@uoc.gr; 5 Department of Surgery, Creta Interclinic Hospital of Heraklion, 71305 Heraklion, Greece; exynos@gmail.com; 6 Department of Medical Oncology, University General Hospital of Heraklion, 71110 Heraklion, Greece; 7 Laboratory of Pathology, University General Hospital of Heraklion, 70013 Heraklion, Greece; tzardi@med.uoc.gr

**Keywords:** colorectal cancer, vitamin D receptors, toll-like receptors, polymorphisms

## Abstract

Vitamin D deficiency has been associated with increased colorectal cancer (CRC) incidence risk and mortality. Vitamin D mediates its action through the binding of the vitamin D receptor (VDR), and polymorphisms of the VDR might explain these inverse associations. The aim of the study was the investigation of the relevance of rs731236; *Thermus aquaticus* I (*Taq*I), rs7975232; *Acetobacter pasteurianus* sub. *pasteurianus* I (*Apa*I), rs2228570; *Flavobacterium okeanokoites* I (*Fok*I) and rs1544410, *Bacillus stearothermophilu*s I (*Bsm*I) polymorphisms of the VDR gene to colorectal carcinogenesis (CRC) and progression. Peripheral blood was obtained from 397 patients with early operable stage II/III (*n* = 202) and stage IV (*n* = 195) CRC. Moreover, samples from 100 healthy donors and 40 patients with adenomatous polyps were also included as control groups. Genotyping in the samples from patients and controls was performed using polymerase chain reaction-restriction fragment length polymorphisms (PCR-RFLP). A significant association was revealed between all four polymorphisms and cancer. Individuals with homozygous mutant (tt, aa, ff or bb) genotypes were more susceptible to the disease (*p* < 0.001). All of the mutant genotypes detected were also significantly associated with stage IV (*p* < 0.001), leading to significantly decreased survival (*p* < 0.001). Moreover, all four polymorphisms were significantly associated with *KRAS* (Kirsten ras oncogene) mutations and Toll-like receptor (TLR2, TLR4 and TLR9) genetic variants. In multivariate analysis, tt, aa and ff genotypes emerged as independent factors associated with decreased overall survival (OS) (*p* = 0.001, *p* < 0.001 and *p* = 0.001, respectively). The detection of higher frequencies of the VDR polymorphisms in CRC patients highlights the role of these polymorphisms in cancer development and progression.

## 1. Introduction

Colorectal cancer (CRC) is the third leading cause of cancer both in men and women [1]. CRC is of major importance to public health, with increased mortality rates worldwide and accounting for 9% of all cancers [1]. CRC is a multifactorial disease and involves complex interactions between environmental and genetic factors [2,3]. However, understanding all of the mechanisms and the associations between these factors is not an easy task. In the past, it has been hypothesized that higher incidence rates of CRC in areas with low sunlight exposure might be attributable to lower levels of vitamin D [4]. Indeed, it has been reported that vitamin D deficiency is a common phenomenon in Saudi Arabia, especially among women [5]. Nowadays, a large number of candidates’ genes have been identified as responsible for their potential role in tumorigenesis. 

The importance of vitamin D for bone health is well established, but lately its role beyond the skeletal system has gained scientific attention [6]. Vitamin D regulates cellular differentiation and proliferation in normal and malignant tissues, regulates proliferation, apoptosis and cell adhesion in tumor cells and modifies tumor angiogenesis, invasion and metastasis, along with reducing oxidative DNA damage [7]. Vitamin D deficiency has been associated with various cancer types [8,9], whereas, increased vitamin D serum levels play a role in decreased colorectal adenoma risk [9]. Vitamin D mediates its action by binding to the vitamin D receptor (VDR), a member of the nuclear receptor superfamily, and is expressed on various cell types, including colorectal epithelial cells, enabling the transactivation of target genes [10,11]. Thus, the VDR gene has been implicated in CRC. Over 60 single nucleotide polymorphisms (SNPs) of the VDR gene, located in the promoter region in exons 2–9, both in their proximity and in the 3’-UTR (3’-untranslated) region, have been studied in relation to cancer occurrence and prognosis [12,13]. However, only a few of them are potentially functional and affect the expression of the VDR gene in relation to CRC risk. These include *Taq*I (rs731236; *Thermus aquaticus* I), located in exon 9 [9,14], *Apa*I (rs7975232; *Acetobacter pasteurianus* sub. *pasteurianus* I) and *Bsm*I (rs1544410, *Bacillus stearothermophilu*s I), located in the intron between exons 8 and 9 [14,15,16] and *Fok*I (rs2228570; *Flavobacterium okeanokoites* I), located in exon 2 [15,17].

To this end, we aimed to investigate four single nucleotide polymorphisms (SNPs)—*Taq*I, *Apa*I, *Fok*I and *Bsm*I—of the VDR gene within patients with sporadic CRC, for the first time in the Greek population, and to evaluate their association with the risk of cancer development and progression. The selection of these SNPs was based on the common VDR SNPs sites examined in other populations in previous genetic epidemiological studies. In addition, the correlation of the expression of these molecules and previously genotyped Toll-like receptor (TLR) variants (TLR2: 196-to-174 del; TLR4: *Asp299Gly* and *Thr399Ile*; TLR9: *T1237C* and *T1486C*) in the same patients’ and controls’ samples [18] was also investigated.

## 2. Results

### 2.1. Patients’ Demographics and Molecular Characteristics

From 09/2003 to 11/2013, 397 patients were recruited in the study and presented newly diagnosed CRC and histologically documented disease. The patients’ characteristics are listed in Table 1. The median age of the patients was 65 years, 246 (62.0%) were males, 202 (50.9%) were of stage II/III, 372 (93.7%) had PS-ECOG (Performance Status according to the Eastern Cooperative Oncology Group) 0–1, 205 (47.4%) had a high tumor grade and 279 (70.3%) had a colon/sigmoid tumor location. Moreover, 230 (58.7%) patients had early operable stage II/III disease, 64 (27.8%) of whom relapsed, whereas 223 (56.2%) patients had stage IV disease, 197 (88.3%) of whom relapsed, as shown in Table 1 and Table S1. 

The VDR gene *Taq*I t allele (silent T → C transition in exon 9), *Apa*I a allele (T → G transition in intron 8), *Fok*I f allele (C → T transition at the junction of intron 1 and exon 2) and *Bsm*I b allele (G → A transition in intron 8) genotypes and allele frequencies were investigated in all 397 patients and are shown in Table 1 and Table S1; whereas *KRAS* (Kirsten ras oncogene) mutations were investigated in 245 patients due to there being no sample availability, shown in Table 1 and Table S1.

### 2.2. Analysis of the VDR Gene Polymorphisms 

**Figure 1:**
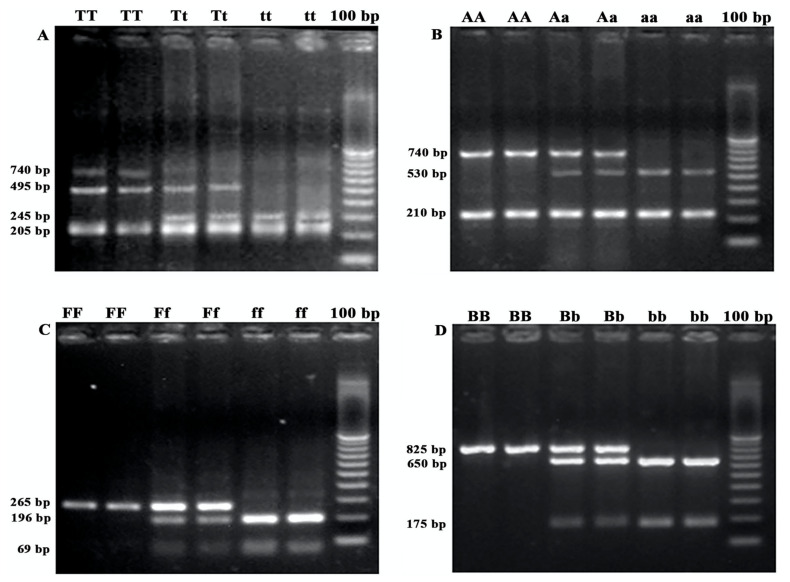
PCR-RFLP representative agarose gels of different SNPs of the (**A**) *Taq*I (TT, Tt, tt), (**B**) *Apa*I (AA, Aa, aa), (**C**) *Fok*I (FF, Ff, ff) and (**D**) *Bsm*I (BB, Bb, bb), respectively.

The VDR gene *Taq*I, *Apa*I, *Fok*I and *Bsm*I polymorphisms amplification products were expected to be 740 bp, 740 bp, 265 bp and 825 bp, respectively. The PCR products were digested by the *Taq*I, *Apa*I, *Fok*I and *Bsm*I enzymes, respectively. Following electrophoresis, 143 (36.0%), 129 (32.5%) and 125 (36.5%) patients presented the homozygous mutant (tt), the heterozygous (Tt) and the wild type (TT) genotypes, respectively, for *Taq*I polymorphisms, as shown in Figure 1A, Table 1 and Table S1. Similarly, 130 (32.7%), 122 (30.7%) and 145 (36.5%) patients presented the homozygous mutant (aa), the heterozygous (Aa) and the wild type (AA) genotypes, respectively, for *Apa*I polymorphisms, as shown in Figure 1B, Table 1 and Table S1. Moreover, 128 (32.2%), 147 (37.0%) and 122 (30.7%) patients presented the homozygous mutant (ff), the heterozygous (Ff) and the wild type (FF) genotypes, respectively, for *Fok*I polymorphisms, as shown in Figure 1C, Table 1 and Table S1. Finally, 131 (33.0%), 191 (48.1%) and 75 (18.9%) patients presented the homozygous mutant (bb), the heterozygous (Bb) and the wild type (BB) genotypes, respectively, for *Bsm*I polymorphisms, as shown in Figure 1D, Table 1 and Table S1.

The results showed that the allelic frequencies of all four polymorphisms were significantly associated with the patient group compared to the control groups (*Taq*I, *p* > 0.001; *Apa*I, *p* > 0.001; *Fok*I, *p* > 0.001; *Bsm*I, *p* > 0.001), thus highlighting the role of these polymorphisms in the disease. More specifically, it was shown that both the healthy donors and the adenomatous polyps controls presented mainly the wild type and the heterozygous genotypes, and this was observed in all four (*Taq*I, *Apa*I, *Fok*I and *Bsm*I) polymorphisms, as shown in Table 2. 

### 2.3. Association of VDR Variants and Disease Stage

Table 2 shows the association observed between the VDR polymorphisms and disease stage of the patients. The tt genotype was more prevalent in stage IV patients, whereas the Tt and TT genotypes were mostly seen in stage II/III patients (62.4% vs. 12.0%, 28.6% vs. 36.1% and 9.0% vs. 51.9%, respectively; *p* < 0.001), as shown Table 2 and Table S1. The aa genotype was more prevalent in stage IV patients, whereas the Aa and AA genotypes were also mostly prevalent in stage II/III patients (61.4% vs. 6.7%, 25.4% vs. 35.6% and 13.2% vs. 57.7%, respectively; *p* < 0.001), as shown in Table 2 and Table S1. Moreover, the ff genotype was also more prevalent in stage IV patients, whereas the Ff and the FF genotypes were mostly met in stage II/III patients (56.6% vs. 10.1%, 30.7% vs. 42.8% and 12.7% vs. 47.1%, respectively; *p* < 0.001), as shown in Table 2 and Table S1. Similarly, the bb genotype was more prevalent in stage IV patients, whereas the Bb and BB genotypes were mostly prevalent in stage II/III patients (49.7% vs. 17.8%, 39.7% vs. 55.8% and 10.6% vs. 26.4%, respectively; *p* < 0.001), as shown in Table 2 and Table S1.

### 2.4. Correlation of VDR and Toll-Like Receptor Variants

The correlation between the different VDR and TLR variants was analyzed and is presented in Table 3. When analyzing the whole group of patients, a statistically significant coexistence was observed both between all the different VDR combinations and between the VDR and TLR genotype combinations, shown in Table 3 and Table S1. When patients were analyzed according to their disease stage, it was observed that stage II/III patients had a significant coexistence of only: *Taq*I-*Fok*I, *p* = 0.005; *Taq*I-*Bsm*I, *p* < 0.001; *Apa*I-*Bsm*I, *p* = 0.014; *Fok*I-*Bsm*I, *p* < 0.001; *Fok*I-*Asp299Gly*, *p* = 0.035; *Fok*I-*Thr399Ile*, *p* = 0.035, as shown in Table 3 and Table S1. Similarly, in stage IV patients, a significant coexistence was observed in: *Taq*I-*Apa*I, *p* < 0.001; *Taq*I-*Bsm*I, *p* < 0.001; *Apa*I-*Bsm*I, *p* < 0.001; *Fok*I-*Apa*I, *p* = 0.001; *Fok*I-*Bsm*I, *p* < 0.001; *Taq*I-T1237C, *p* = 0.042; *Taq*I-T1486C, *p* = 0.042; *Fok*I-*Asp299Gly*, *p* = 0.037; *Fok*I-*Thr399Ile*, *p* = 0.037, as shown in Table 3 and Table S1.

### 2.5. Association of TaqI, ApaI, FokI and BsmI Variants and KRAS Status

The VDR variants and *KRAS* statuses of the CRC patients presented a significant association, as shown in Table 4. The tt genotype was more prevalent in patients with a mutant *KRAS* status, whereas the Tt and the TT alleles were mostly seen in *KRAS* wild type patients (53.8% vs. 39.0%, 24.0% vs. 34.8% and 22.1% vs. 26.2%, respectively, *p* = 0.041). The aa genotype was also more frequent in *KRAS* mutants, whereas Aa and AA genotypes were more frequent in patients with *KRAS* wild type status (55.8% vs. 36.9%, 19.2% vs. 34.8% and 25.0% vs. 28.4%, respectively; *p* = 0.007). Similarly, the ff genotype was more prevalent in *KRAS* mutants, whereas the Ff and FF genotypes were more frequent in *KRAS* wild type patients (59.6% vs. 27.7%, 25.0% vs. 42.6% and 15.4% vs. 29.8%, respectively; *p* < 0.001). Finally, the bb genotype was also more frequent in *KRAS* mutants, whereas Bb and BB genotypes were more frequent in patients with *KRAS* wild type status (65.4% vs. 27.7%, 24.0% vs. 55.3% and 10.6% vs. 17.0%, respectively; *p* < 0.001), as shown in Table 4 and Table S1. 

### 2.6. Association of VDR Variants and Clinical Outcome

**Figure 2:**
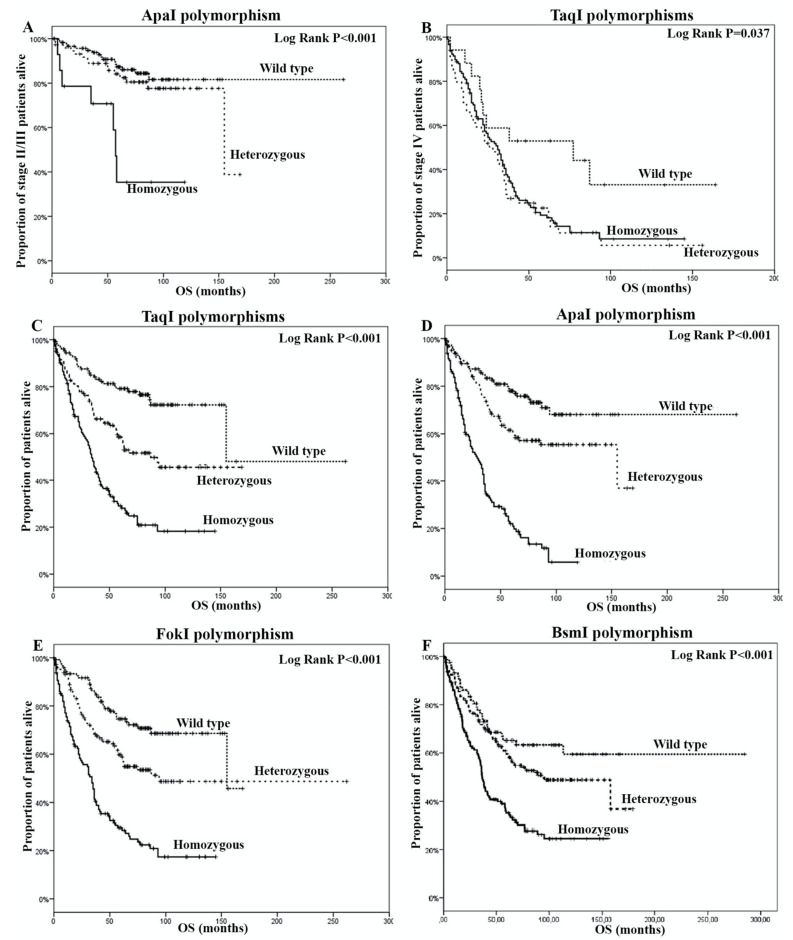
Overall survival according to the detection of (**A**) *Apa*I polymorphisms and (**B**) *Taq*I polymorphisms in stage II/III patients, and (**C**) *Taq*I polymorphisms, (**D**) *Apa*I polymorphisms, (**E**) *Fok*I polymorphisms and (**F**) *Bsm*I polymorphisms in stage IV patients.

Sixty-four (27.8%) adjuvant and 197 (49.7%) metastatic patients presented a disease progression following their adjuvant and first-line treatment, respectively, as shown in Table S1. The median disease-free survival (DFS) was 19 months (95% confidence interval (CI): 15.5–22.5) and the median overall survival (OS) was 155 months (95% CI: 59.1–250.9), respectively for stage II/III patients. According to the presence of different VDR genotypes, no significant differences were observed in DFS, whereas only a significant shorter OS in patients with the aa genotype (*p* < 0.001) was observed, as shown in Figure 2A. 

For the case of stage IV patients, the median progression-free survival (PFS) was 8 months (95% CI: 7.1–8.9) and the median OS was 31 months (95% CI: 25.2–36.8), respectively, as shown in Table S1. Again, there was no difference in PFS, according to the presence of different VDR genotypes, whereas only a significant decrease in OS in patients with *Taq*I homozygous mutant or heterozygous alleles (*p =* 0.037) was observed, as shown in Figure 2B. The analysis of all patients presented a median OS of 75 months (95% CI: 56.8–93.2) prevailing a significantly decreased OS in patients with *Taq*I, *Apa*I, *Fok*I and/or *Bsm*I homozygous mutant alleles (*p* < 0.001, *p* < 0.001, *p* < 0.001 and *p* < 0.001, respectively), as shown in Figure 2C–F and Table S1.

### 2.7. Univariate and Multivariate Analysis Cox Regression Analysis

Univariate analysis revealed that PS (ECOG) and TLR2 polymorphisms were significantly associated with a shorter PFS and PS (ECOG) tumor grade, and all TLR and VDR polymorphisms were significantly associated with shorter OS, as shown in Table 5. In multivariate analysis, adjusting for these factors, PS (ECOG) and TLR2 polymorphisms emerged as independent factors associated with decreased PFS (HR: 1.6, 95% CI: 1.0–2.6, *p =* 0.04 and HR: 2.2, 95% CI: 1.2–4.0, *p =* 0.013, respectively). Moreover, PS (ECOG), tumor grade, TLR2 196-to-174 del, *Taq*I, *Apa*I and *Fok*I variants emerged as independent factors associated with decreased OS (HR: 3.7, 95% CI: 2.4–5.9, *p <* 0.001; HR: 1.8, 95% CI: 0.7–5.1, *p <* 0.001; HR: 2.1, 95% CI: 1.3–3.4, *p =* 0.003; HR: 1.3, 95% CI: 1.0–1.5, *p =* 0.029; HR: 1.6, 95% CI: 1.3–2.0, *p <* 0.001; HR: 1.4, 95% CI: 1.1–1.8, *p =* 0.005), as shown in Table 5.

## 3. Discussion

Undoubtedly, the risk of sporadic CRC has been linked to environmental and genetic factors [2,19] and more recently to gut microbiota. The microbiota influences both human health and disease by affecting the development of the host immune system and by maintaining homeostasis to influence diseases and allergies that cannot simply be parsed into strict pathogenesis and commensalism [20,21]. Chronic infection and inflammation are the most important epigenetic factors contributing to tumorigenesis and tumor progression [22]. Moreover, vitamin D deficiency has been implicated, among other diseases and metabolic syndromes, in human malignancies [23,24]. Since vitamin D mediates its action by binding to the VDR gene, the suboptimal responsiveness of the VDR can be manifested as vitamin D deficiency. Furthermore, the VDR gene, as part of innate immunity, is responsible for the prevention and elimination of infection and the determination of the gut microbiome [25,26,27]. Thus, polymorphisms in the human VDR gene may play an important role in the structure of the gut microbiome. Interestingly, it has been reported previously that VDR conditional knockout (*vdr*^ΔIEC^) in the intestinal epithelial or low intestinal VDR protein levels may lead to dysbiosis [25] and reduced autophagy, accompanied by a reduction in *ATG16L1* (autophagy-related 16 like 1), an inflammatory bowel disease risk gene [27]; whereas, the absence of intestinal VDR leads to a susceptibility to colon cancer via reducing JAK/STAT (Janus kinases/signal transducer and activator of transcription proteins) signaling, which is a pathway with a critical role in intestinal and microbial homeostasis and in dampening inflammatory responses [27]. Therefore, the vitamin D/VDR pathway may significantly influence homeostasis, signaling between the microbiota and host in intestinal inflammation and tumorigenesis [27]. It is worth mentioning that, despite the fact that the VDR gene polymorphisms (*Taq*I, *Apa*I, *Fok*I and *Bsm*I) are considered to be non-functional, they might be linked to other functional polymorphisms elsewhere in the VDR gene, thus participating in a more complex gene network, enhancing or inhibiting the expression of VDR target genes. Such VDR gene polymorphisms are likely to affect transcriptional regulation, mRNA stability or protein translational efficiency, thus affecting the structure and functioning of VDR protein [9,28,29,30]. It has also been suggested that VDR gene polymorphisms define differential transcriptional VDR activity or mRNA stability in vitro [31]. Moreover, vitamin D is considered as an immunomodulatory molecule, which can modulate cytokine responses through T cells, thus representing an important link between TLR activation and innate immunity against microorganisms [32,33]. Others have described the TLR activation of human macrophages upregulating VDR gene expression, leading to the induction of cathelicidin and the consequent killing of intracellular *Mycobacterium tuberculosis* [34]. Moreover, the authors further analyzed *Cyp27B1* (cytochrome P450 family 27 subfamily B member 1), which catalyzes the conversion of the inactive provitamin D3 hormone into its active form, and it was found to be significantly upregulated. Furthermore, the VDR and *Cyp27B1* in monocytes and macrophages were also found to be upregulated. Thus, the authors concluded that the TLR induces the upregulation of the VDR and *Cyp27B1* gene expressions in such cell types [34]. They also performed functional tests on the VDR and it was demonstrated that the VDR is functional in primary human monocytes and, when activated, it triggers the stimulation of antimicrobial peptides. Finally, the authors demonstrate that TLR activation in monocytes might lead to the activation of a microbicidal pathway, which depends on the production and action of vitamin D through VDR. Overall, the authors provided a potential explanation of how vitamin D may act as a key link between TLR activation and antimicrobial responses in innate immunity [34]. The aim of the current study was to evaluate the detection of VDR (*Taq*I, *Apa*I, *Fok*I and *Bsm*I) polymorphisms in adjuvant and metastatic CRC patients.

A number of studies on VDR polymorphisms have been performed in patients with sporadic CRC. Some studies reported contradictory results between various VDR genetic variants and CRC [35,36], or even no association [13,37], probably due to limitations such as the recruitment of patients with different characteristics, such as ethnicity, among these studies and the small sample size. However, many of these studies presented significant associations [35,38]. Indeed, in a meta-analysis conducted by Serrano et al., [39] the authors presented a significant increased risk for CRC in the presence of tt and aa genotypes, with strong frequency variations present among different ethnic groups. Moreover, Gandini et al., [24] reviewed 79 studies with more than 52,000 cancer cases, including CRC patients, and 62,000 controls, and demonstrated that *Bsm*I, *Fok*I and *Taq*I polymorphisms are associated with CRC. Additional studies reported that the bb genotype of the *Bsm*I polymorphism are more susceptible to CRC, whereas BB, Bb, TtFf and TTFf genotypes are significantly associated with a decreased risk of CRC [14,35,40,41]. Our results are in accordance with these findings. In fact, we demonstrate a higher frequency of the tt, aa, ff and bb genotypes in CRC patients compared to the control groups, thus highlighting the role of these polymorphisms in colorectal carcinogenesis. Moreover, higher frequencies of the tt, aa, ff and bb genotypes were detected in metastatic CRC patients compared to stage II/III patients, emphasizing the role of these polymorphisms in CRC progression and in patients’ overall survival. 

The Ras/MAPK (Ras/mitogen activated protein kinases) pathway and its continuous activation, due to mutations presented in codon 12 of the *KRAS* gene, plays an important role in treatment resistance in patients with various carcinomas, including CRC [42]. To this end, we also aimed to associate the frequency of VDR polymorphisms in patients with different *KRAS* statuses. Despite the fact that approximately 40% of the enrolled patients were not evaluated for their *KRAS* statuses, a significant association was demonstrated between the tt, aa, ff and bb genotypes with the *KRAS* mutant patients.

The TLR pathway increases the risk of colitis-associated CRC due to commensal gut microbiota [43,44]. TLRs play an important role in immunity and are expressed in various cell types, including tumor cells [45,46]. Since TLR polymorphisms have been associated with changes in susceptibility to many diseases, including cancers [47] and TLRs promote the survival of cancer cells [48], we also correlated the coexistence of previously genotyped TLR (TLR2, TLR4 and TLR9) polymorphisms [18] with the VDR polymorphisms. It has been previously reported that human TLRs are considered to be regulators in vitamin D/VDR signaling. In humans, when a pathogen is detected by TLRs, gene expressions of VDR and *Cyp27B1* are induced [34,49]. 

Herein, we demonstrated that both TLR [18] and VDR polymorphisms are associated with an increased risk of CRC development and progression, with an impact on patients’ survival, and also demonstrated a significant correlation between TLR and VDR gene polymorphisms. 

To our knowledge, this is the first time VDR gene polymorphisms have been investigated in the Greek population to evaluate their role in CRC risk and patients’ survival. The strength of the present study includes careful clinical and epidemiological data collection in combination with genotyping. Such data collection allowed us to investigate gene-to-gene or other interactions. Moreover, the relatively large sample size and the 10-year follow-up of the enrolled patients provided enough strength to discriminate significant interactions that have an impact on CRC risk and the survival of the patients. An additional strength is that the enrolled cases and controls were from the same ethnicity and were age and gender matched. A limitation of the study is the lack of vitamin D measurements in the plasma or sera of the enrolled patients and controls, due to the retrospective nature of the study. However, despite this limitation, the results of this study provide significant information regarding the association between *Taq*I, *Apa*I and *Fok*I polymorphisms and susceptibility to CRC and its progression. 

## 4. Materials and Methods 

### 4.1. Patients’ Population 

In total, 397 patients with colon adenocarcinoma were enrolled in the study, between 2003 and 2013, from the Department of Medical Oncology, University Hospital of Heraklion. 

### 4.2. Ethics Approval and Consent to Participate

The study has been approved by the Ethics Committee/Institutional Review Board of the University Hospital of Heraklion (Number 7302/19-8-2009), and all patients signed a written informed consent form for their participation. All of the procedures performed were in accordance with the ethical standards of the institutional and/or national research committee and the 1964 Helsinki declaration, and its later amendments or comparable ethical standards

### 4.3. Blood and Tissue Samples from Control Groups

In parallel to patients’ samples, blood samples and formalin-fixed paraffin embedded (FFPE) tissues were also obtained from 100 healthy blood donors and from 40 patients with colon adenomas in the absence of CRC disease, respectively, which were used as controls in the study. 

### 4.4. Genomic DNA Extraction

Peripheral blood mononuclear cells (PBMC) from all individuals (patients and healthy donors) were obtained using the Ficoll–Hypaque density gradient (*d* = 1077 g/mL; Sigma-Aldrich, GmbH, Darmstadt, Germany), as described previously [18]. Representative formalin fixed paraffin embedded (FFPE) specimens from the primary tumor were examined by an experienced pathologist and the appropriate area for microdissection was defined. Microdissection and malignant cells collection were performed using a piezoelectric microdissector (Eppendorf, Hamburg, Germany), as described previously [50]. 

The DNA extraction of all samples was performed using the MasterPure™ Complete DNA and RNA Purification Kit (Epicenter, Madison, WI, USA), according to the manufacturer’s instructions. NanoDrop ND-1000, version 3.3 (ThermoFisher Scientific, Waltham, MA, USA) was used for DNA quantification. 

### 4.5. VDR and TLR Genotyping 

For the genotyping of the SNPs at the *TaqI*, *ApaI*, *FokI* and *BsmI* positions of the VDR gene, polymerase chain reaction (PCR) and restriction fragment length polymorphism (RFLP) methods were used. The sequences of the primers used for the PCR amplification of the fragments are provided in Table 6. Allele types, SNP reference numbers and PCR conditions for all the analyzed polymorphisms are shown in Table 7. 

*Taq*I (Minotech Biotechnology, IMBB-FORTH, Heraklion, Greece), *Apa*I (ThermoFisher Scientific, MA, USA), *Fok*I (ThermoFisher Scientific) and *Bsm*I (ThermoFisher Scientific) restriction enzymes were used to digest the amplified products of the VDR gene, according to manufacturer’s instructions. Briefly, 10 μL of each related PCR product was mixed with 1 μL of each restriction enzyme and 2 μL of 10× buffers. Diethyl pyrocarbonate (DEPC) treated water was added to a final volume of 20 μL (for *Taq*I) or 30 μL (for *Apa*I, *Fok*I and *Bsm*I). After incubation at 65 °C for 15 min (*Taq*I) or at 37 °C for 5 min (*Apa*I, *Fok*I and *Bsm*I), the restriction fragments were separated by electrophoresis on a 2% agarose gel, stained with Sybr Safe DNA Gel Stain (ThermoFisher Scientific), and were visualized with the AlphaImager ultraviolet transilluminator (Alpha Innotech Corp., San Leandro, CA, USA). The usual nomenclature for restriction fragment length polymorphism alleles was used in this study [51,52]. The lowercase (t, a, f, b) alleles represent the presence of the restriction site and the uppercase alleles (T, A, F, B) represent the absence of the restriction site.

Accordingly, TLR genotyping was performed as previously described by our group [18]. In brief, the determination of TLR2 196-to-174 Ins*/*Del polymorphism was performed by PCR, whereas the determinations of TLR4 and TLR9 were performed by PCR-RFLP. The PCR and PCR-RFLP conditions and primers sets that were used have been previously reported by our group [18]. 

### 4.6. KRAS Mutational Analysis

*KRAS* mutational analysis was performed by Sanger sequencing after the PCR amplification of *KRAS* exon 2. The PCR conditions and the primers set that was used have been previously reported by our group [53].

### 4.7. Study Design and Statistics

The current study is a retrospective, single institution study which aimed to investigate the VDR gene polymorphisms in CRC patients before the initiation of any treatment. Disease-free survival (DFS), progression-free survival (PFS) and overall survival (OS) were calculated as previously described [18]. In brief, DFS was calculated from the date of surgery to the date of disease recurrence, PFS was calculated from the date of diagnosis to documented disease progression or death from any cause, and OS was calculated from the date of diagnosis to the date of death, from any cause. Laboratory analysis was carried out blind to clinical data, and statistical analysis was based on contingency tables, including the calculations of hazard ratios (HR) and 95% CI, as previously described [18]. Statistical significance was set at *p* = 0.05.

## 5. Conclusions

In conclusion, the results of the present study highlight the significant role of VDR polymorphisms in carcinogenesis, disease progression and patients’ survival. Our data also showed a correlation between TLR and VDR expression and an increased impact on patients’ survival. Based on the present results, therapies targeting the activity of VDRs, including the modulation of the TLR/VDR pathways, might provide new approaches to the management of CRC. 

## Figures and Tables

**Table 1:** Patients’ demographics and characteristics.

Characteristics	Frequency (*n* = 397)	%
**Age (range)**	65 (18–88)	
<70	260	65.5
≥70	137	34.5
**Gender**		
Male	246	62
Female	151	38
**Stage**		
IIA–IIIC	202	50.9
IV	195	49.1
**Location**		
Colon/Sigmoid	279	70.3
Rectum	118	29.7
**PS (ECOG)**		
0–1	372	93.7
≥2	25	6.3
**Surgery**		
Yes	347	87.8
No	50	12.2
**Radiotherapy**		
Yes	80	20.4
No	313	79.6
**Adjuvant Treatment**		
Yes	230	58.7
No	162	41.4
**First Line Treatment**		
Yes	223	56.2
No	174	46.8
Grade		
High	205	47.4
Low	228	52.6
*Taq* **I (T to C)**		
Wt (TT)	125	31.5
Hetero (Tt)	129	32.5
Homo (tt)	143	36
*Apa* **I (T to G)**		
Wt (AA)	145	36.5
Hetero (Aa)	122	30.7
Homo (aa)	130	32.7
*Fok* **I (C to T)**		
Wt (FF)	122	30.7
Hetero (Ff)	147	37
Homo (ff)	128	32.2
*Bsm* **I (T to C)**		
Wt (BB)	75	18.9
Hetero (Bb)	191	48.1
Homo (bb)	131	33
KRAS		
Mutant	104	42.4
Wild type	141	57.6
ND	152	

**Table 2:** Association of VDR polymorphisms between patients and control groups (healthy donors and adenomatous polyps) and between different patients’ stages (II/III and IV).

SNP	Genotype	Patient No (%)	Healthy No (%)	Adenomatous Polyps No (%)	*p*-Value	Stage II/III No (%)	Stage IV No (%)	*p*-Value
*Taq*I	Wt (TT)	125 (31.5)	71 (71)	24 (60.0)	<0.001	108 (51.9%)	17 (9.0%)	<0.001
	Hetero (Tt)	129 (32.5)	26 (26.0)	11 (27.5)		75 (36.1%)	54 (28.6%)	
	Homo (tt)	143 (36.0)	3 (3.0)	5 (12.5)		25 (12.0%)	118 (62.4%)	
*Apa*I	Wt (AA)	145 (36.5)	56 (56.0)	22 (55.0)	<0.001	120 (57.7%)	25 (13.2%)	<0.001
	Hetero (Aa)	122 (30.7)	40 (40.0)	17 (42.5)		74 (35.6%)	48 (25.4%)	
	Homo (aa)	130 (32.7)	4 (4.0)	1 (2.5)		14 (6.7%)	116 (61.4%)	
*Fok*I	Wt (FF)	122 (30.7)	55 (55.0)	21 (52.5)	<0.001	98 (47.1%)	24 (12.7%)	<0.001
	Hetero (Ff)	147 (37.0)	40 (40.0)	16 (40.0)		89 (42.8%)	58 (30.7%)	
	Homo (ff)	128 (32.2)	5 (5.0)	3 (7.5)		21 (10.1%)	107 (56.6%)	
*Bsm*I	Wt (BB)	75 (18.9)	55 (55.0)	13 (32.5)	<0.001	55 (26.4%)	20 (10.6%)	<0.001
	Hetero (Bb)	191 (48.1)	43 (43.0)	24 (60.0)		116 (55.8%)	75 (39.7%)	
	Homo (bb)	131 (33.0)	2 (2.0)	3 (7.5)		37 (17.8%)	94 (49.7%)	

**Table 3:** Correlation of various VDR and TLR polymorphisms. All values presented correspond to *p*-values.

All patients		***Taq*I**	***Apa*I**	***Fok*I**	***Bsm*I**	**TLR4 (*Asp299Gly*)**	**TLR4 (*Thr399Ile*)**	**TLR9 (*T1237C*)**	**TLR9 (*T1486C*)**	**TLR2 (196-to-174 del)**
	*Taq*I	-	0.000	0.000	0.000	0.000	0.000	0.000	0.000	0.000
	*Apa*I	0.000	-	0.000	0.000	0.000	0.000	0.000	0.000	0.000
	*Fok*I	0.000	0.000	-	0.000	0.000	0.000	0.002	0.002	0.000
	*Bsm*I	0.000	0.000	0.000	-	0.004	0.004	0.026	0.026	0.009
Adjuvant		*Taq*I	*Apa*I	*Fok*I	*Bsm*I	TLR4 (*Asp299Gly*)	TLR4 (*Thr399Ile*)	TLR9 (*T1237C*)	TLR9 (*T1486C*)	TLR2 (196-to-174 del)
	*Taq*I	-	0.301	0.005	0.000	0.803	0.803	0.388	0.388	0.346
	*Apa*I	0.301	-	0.588	0.014	0.733	0.733	0.944	0.944	0.148
	*Fok*I	0.005	0.588	-	0.000	0.035	0.035	0.808	0.808	0.272
	*Bsm*I	0.000	0.014	0.000	-	0.966	0.966	0.905	0.905	0.266
Metastatic		*Taq*I	*Apa*I	*Fok*I	*Bsm*I	TLR4 (*Asp299Gly*)	TLR4 (*Thr399Ile*)	TLR9 (*T1237C*)	TLR9 (*T1486C*)	TLR2 (196-to-174 del)
	*Taq*I	-	0.000	0.203	0.000	0.874	0.874	0.042	0.042	-
	*Apa*I	0.000	-	0.001	0.000	0.295	0.295	0.023	0.023	-
	*Fok*I	0.203	0.001	-	0.000	0.037	0.037	0.973	0.973	-
	*Bsm*I	0.000	0.000	0.000	-	0.703	0.703	0.814	0.814	-

**Table 4:** Association of VDR gene polymorphisms and *KRAS* status.

*KRAS*				
SNP	Genotype	Wt (%)	Mutant (%)	*p*-Value
*Taq*I	Wt (TT)	37 (26.2)	23 (22.1)	0.041
	Hetero (Tt)	49 (34.8)	25 (24.0)	
	Homo (tt)	55 (39.0)	56 (53.8)	
*Apa* **I**	Wt (AA)	40 (28.4)	26 (25.0)	0.007
	Hetero (Aa)	49 (34.8)	20 (19.2)	
	Homo (aa)	52 (36.9)	58 (55.8)	
*Fok*I	Wt (FF)	42 (29.8)	16 (15.4)	<0.001
	Hetero (Ff)	60 (42.6)	26 (25.0)	
	Homo (ff)	39 (27.7)	62 (59.6)	
*Bsm* **I**	Wt (BB)	24 (17.0)	11 (10.6)	<0.001
	Hetero (Bb)	78 (55.3)	25 (24.0)	
	Homo (bb)	39 (27.7)	68 (65.4)	

**Table 5:** Univariate and multivariate Cox Regression analysis.

	Univariate Analysis				Multivariate Analysis			
	PFS		OS		PFS		OS	
	HR (95% CI)	*p*-Value	HR (95% CI)	*p*-Value	HR (95% CI)	*p*-Value	HR (95% CI)	*p*-Value
Gender (Male vs. Female)	1.0 (0.8–1.4)	0.986	0.9 (0.7–1.2)	0.411	-	-	-	-
Histology (Colon/Sigmoid vs. Rectum)	1.2 (0.9–1.6)	0.267	1.2 (0.9–1.7)	0.172	-	-	-	-
PS ECOG (≥2 vs. 0–1)	2.6 (1.6–4.2)	<0.001	4.4 (2.8–6.9)	<0.001	1.6 (1.0–2.6)	0.04	3.7 (2.4–5.9)	<0.001
Age (≥70 vs. <70)	1.1 (0.8–1.4)	0.652	1.3 (1.0–1.8)	0.06	-	-	-	-
Grade (High vs. Low)	1.4 (0.6–3.4)	0.493	4.4 (1.8–11.0)	0.001	-	-	1.8 (0.7–5.1)	<0.001
*KRAS*	1.0 (0.8–1.4)	0.787	1.2 (0.8–1.6)	0.362	-	-	-	-
TLR2 196-to-174 del (homozygous mutant vs. others)	2.1 (1.2–3.9)	0.016	3.7 (2.4–5.9)	<0.001	2.2 (1.2–4.1)	0.013	2.1 (1.3–3.4)	0.003
TLR4-Asp299Gly (homozygous mutant vs. others)	1.2 (0.9–1.7)	0.259	2.3 (1.7–3.3)	<0.001	-	-	1.4 (1.0–1.9)	0.095
TLR4-Thr399Ile (homozygous mutant vs. others)	1.2 (0.9–1.7)	0.259	2.3 (1.7–3.3)	<0.001	-	-	1.4 (1.0–1.9)	0.095
TLR9-T1237C (homozygous mutant vs. others)	1.0 (0.7–1.4)	0.928	1.7 (1.3–2.3)	<0.001	-	-	1.1 (0.8–1.6)	0.336
TLR9-T1486C (homozygous mutant vs. others)	1.0 (0.7–1.4)	0.928	1.7 (1.3–2.3)	<0.001	-	-	1.1 (0.8–1.6)	0.336
*Taq*I (homozygous mutant vs. others)	1.2 (0.9–1.2)	0.783	2.2 (1.8–2.7)	<0.001	-	-	1.3 (1.0–1.6)	0.029
*Apa*I (homozygous mutant vs. others)	1.1 (0.9–1.3)	0.46	2.6 (2.1–3.1)	<0.001	-	-	1.6 (1.3–2.0)	<0.001
*Fok*I (homozygous mutant vs. others)	1.1 (0.9–1.2)	0.768	2.2 (1.9–2.6)	<0.001	-	-	1.4 (1.1–1.8)	0.005
*Bsm*I (homozygous mutant vs. others)	0.1 (0.9–1.3)	0.616	1.7 1.4–2.2)	<0.001	-	-	1.0 (0.7–1.6)	0.644

**Table 6:** PCR primers designed to amplify fragments harboring the VDR single nucleotide polymorphisms.

SNP	Primer	Sequence	Fragment Size
***Taq*** **I**	Forward	5′-CAG AGC ATG GAC AGG GAG CAA-3′	740 bp uncleaved
	Reverse	3′-GCA ACT CCT CAT GGC TGA GGT CTC-5′	495 bp, 245 bp or 290 bp, 245 bp, 205 bp
***Apa*** **I**	Forward	5′-CAG AGC ATG GAC AGG GAG CAA-3′	740 bp uncleaved
	Reverse	3′-GCA ACT CCT CAT GGC TGA GGT CTC-5′	530 bp, 210 bp
***Fok*** **I**	Forward	5′-AGC TGG CCC TGG CAC TGA CTC TGC TCT-3′	265 bp uncleaved
	Reverse	3′-ATG GAA ACA CCT TGC TTC TTC TCC CTC-5′	196 bp, 69 bp
***Bsm*** **I**	Forward	5′-CAA CCA AGA CTA CAA GTA CCG CGT CAG TGA-3′	825 bp uncleaved
	Reverse	3′-AAC CAG CGG GAA GAG GTC AAG GG-5′	650 bp, 175 bp

**Table 7:** Allele types, single nucleotide polymorphism (SNP) reference numbers and PCR conditions.

SNP	Allele Type	Ref. Number	PCR Conditions
***Taq*** **I**	t allele: silent T → C transition in exon 9	rs731236	Initial heating at 94 °C for 2 min, followed by 30 cycles of denaturing (at 94 °C for 30 s), annealing (at 57 °C for 30 s) and chain extension (at 72 °C for 1 min), followed by a final extension step at 72 °C for 7 min
***Apa*** **I**	a allele: T → G transition in intron 8	rs7975232	Initial heating at 94 °C for 2 min, followed by 30 cycles of denaturing (at 94 °C for 30 s), annealing (at 57 °C for 30 s) and chain extension (at 72 °C for 1 min), followed by a final extension step at 72 °C for 7 min
***Fok*** **I**	f allele: C → T transition at the junction of intron 1 and exon 2	rs2228570	Initial heating at 95 °C for 2 min, followed by 35 cycles of denaturing (at 94 °C for 30 s), annealing (at 58 °C for 30 s) and chain extension (at 72 °C for 1 min), followed by a final extension step at 72 °C for 7 min
***Bsm*** **I**	b allele: G → A transition	rs1544410	Initial heating at 95 °C for 2 min, followed by 35 cycles of denaturing (at 94 °C for 30 s), annealing (at 60 °C for 30 s) and chain extension (at 72 °C for 1 min), followed by a final extension step at 72 °C for 7 min

## Supplementary Materials

The following are available online at https://www.mdpi.com/2072-6694/12/6/1379/s1, Table S1: Research raw data.
